# Reliability and validity of specific quality of life assessment questionnaires related to chronic venous insufficiency: a systematic review

**DOI:** 10.1590/1677-5449.202102292

**Published:** 2022-10-24

**Authors:** Igor Lucas Geraldo Izalino de Almeida, Pedro Henrique Scheidt Figueiredo, Whesley Tanor Silva, Vanessa Amaral Mendonça, Ana Cristina Rodrigues Lacerda, Vanessa Pereira Lima, Alessandra de Carvalho Bastone, Henrique Silveira Costa

**Affiliations:** 1 Universidade Federal dos Vales do Jequitinhonha e Mucuri – UFVJM, Diamantina, MG, Brasil.

**Keywords:** venous insufficiency, quality of life, validation study, patient health questionnaire, insuficiência venosa, qualidade de vida, estudo de validação, questionário de saúde do paciente

## Abstract

This systematic review aimed to discuss the main findings regarding the reliability and validity of health-related quality of life questionnaires for chronic venous insufficiency. Searches were performed on the MEDLINE, CINAHL, Web of Science, LILACS, and Scopus databases. The search terms used were related to “venous insufficiency”, and “quality of life”. The CIVIQ-20 and CIVIQ-14 instruments had adequate internal consistency and both were able to discriminate disease severity. The VEINES-QoL showed adequate internal consistency but was not able to discriminate disease severity. Most studies did not demonstrate a correlation between VEINES-QoL and the mental component of the SF-36. The AVVQ had inadequate reliability but its validity was also doubtful when compared to the SF-36. The VARIShort demonstrated good internal consistency, reproducibility, and validity, but only the original study was included. For venous leg ulcers, the CCVUQ showed adequate reliability and validity when compared to VLU-QoL.

## Resumo

Esta revisão sistemática objetivou discutir os principais achados sobre a confiabilidade e validade dos questionários de qualidade de vida relacionada à saúde na insuficiência venosa crônica. A busca foi realizada nas bases de dados MEDLINE, CINAHL, Web of Science, LILACS e Scopus. Os termos de busca foram relacionados a “insuficiência venosa” e “qualidade de vida”. O *Chronic Lower Limb Venous Insufficiency Questionnaire* (CIVIQ)-20 e o CIVIQ-14 apresentaram consistência interna adequada e foram capazes de discriminar a gravidade da doença. O *Venous Insufficiency Epidemiological and Economic Study – Quality of Life* (VEINES-QoL) apresentou consistência interna adequada, mas não foi capaz de discriminar a gravidade da doença. A maioria dos estudos não demonstrou associação do VEINES-QoL com o componente mental do *Short Form Health Survey* (SF-36). O *Aberdeen Varicose Vein Questionnaire* (AVVQ) apresentou confiabilidade inadequada e validade duvidosa quando comparado ao SF-36. O VARIShort demonstrou consistência interna, reprodutibilidade e validade boas, mas apenas o estudo original foi incluído. Na úlcera venosa, o *Charing Cross Venous Ulcer Questionnaire* (CCVUQ) apresentou confiabilidade e validade adequadas quando comparado ao *Venous Leg Ulcer Quality of Life* (VLU-QoL).

## INTRODUCTION

Chronic venous insufficiency (CVI) is a health condition caused by venous valve incompetence, usually associated with calf pump dysfunction.^1^^-^3 The signs and symptoms of CVI span a wide spectrum of severity, ranging from asymptomatic to active and recurrent venous leg ulcers.^4^ Severity levels can be assessed according to Clinical, Etiological, Anatomical, and Pathophysiological (CEAP) class, stratifying patients by presence of telangiectasias or reticular veins (C1), varicose veins (C2), edema (C3), trophic abnormalities (C4), healed ulcer (C5), and active ulcer (C6).^5^

Chronic venous insufficiency prevalence rates are high and CVI affects about 25% of the general population.^6^ Thus, its treatment generates significant costs for patients and healthcare systems.^7^ Furthermore, studies have shown that when patients with CVI are compared to healthy individuals, they have reduced lower limbs muscle strength^8^ and ankle range of motion,^9^ changes in gait and balance,^2^ and, consequently, worse health-related quality of life (HRQoL).^10^

A previous study recommended use of HRQoL assessment in clinical monitoring and patient management^11^ to guarantee analysis of the true impact of diseases on daily life. The HRQoL questionnaires used in several different cardiovascular pathologies and for long-term patient follow-up have emerged as markers of clinical improvement and offer a means for stratification of patient risk.^12^ In the setting of CVI, HRQoL questionnaires have been used as a valuable tool to improve decision-making, such as deciding on referral to specialized centers.^13^ Many studies have addressed assessment of HRQoL in this population and also the effect of interventions on HRQoL.^14^^-^16

However, assessment of HRQoL in patients with CVI is complex, since its clinical expression ranges from esthetic factors to functional components.^17^ There is therefore a need to critically discuss the psychometric properties, i.e., reliability, validity, and responsiveness, of the specific questionnaires available to ensure proper use. Reliability and validity stand out among the psychometric properties evaluated by researchers and professionals.^18^^,^^19^ Reliability refers to an instrument’s ability to reproduce consistent results, involving aspects such as coherence, stability, precision, equivalence, and homogeneity.^20^ On the other hand, validity is not a characteristic of the instrument, but rather it refers to the instrument’s ability to measure exactly what it proposes to measure in a defined population.^21^^,^^22^ Careful analysis of reliability and validity is therefore useful for routine clinical practice.

Questionnaires must provide valid data for a specific population. For example, a questionnaire may be valid for assessing the HRQoL of patients with peripheral arterial disease, but not for patients with CVI. In addition, measurements must provide scientifically robust and reliable results. It is therefore necessary to determine the reliability and validity of HRQoL questionnaires before they are administered. Data on the aforementioned psychometric properties of HRQoL questionnaires in the context of CVI are scarce and remain unclear.^23^ Determining these psychometric properties should assist in the choice of which questionnaires are appropriate for specific target populations. The present study aimed to critically discuss the main findings available in the literature on the reliability and validity of disease-specific HRQoL questionnaires available for patients with CVI.

## METHODS

### Study design

This study is a systematic review of cross-sectional, cohort, or case-control studies. The protocol was registered on the Open Science framework (protocol available at https://osf.io/fsuwj/) and written following the guidelines of the Preferred Reporting Items for Systematic Reviews and Meta-Analyses (PRISMA) statement^24^ and the Cochrane recommendations.^25^

### Search strategy and study selection

Searches were conducted on the Medical Literature Analysis and Retrieval System Online (MEDLINE), the Cumulative Index of Nursing and Allied Health Literature (CINAHL), the Web of Science, Latin American & Caribbean Health Sciences Literature (LILACS), and Scopus, with no language or date restrictions, from database inception to July 2021. Searches were conducted independently by 2 authors (ILGIA and WTS) from April to July 2021. Disagreements were resolved by a third reviewer (HSC). Search terms were related to “venous insufficiency” and “quality of life”. After searching the databases, the references retrieved were exported to an Endnote® file and duplicates were removed (duplicates not found by the software were deleted manually). The following strategy was used for the PubMed search: (“venous insufficiency” OR “venous disease” OR “Chronic venous disease”) AND (“quality of life” OR “health-related quality of life”) and was modified as appropriate for each of the other databases.

### Eligibility criteria

Eligibility criteria were studies that evaluated the reliability and validity of HRQoL questionnaires in patients with CVI, regardless of the degree of severity according to CEAP classification. Thus, studies of patients of both sexes, of any age, from any health institution were considered eligible. Potentially eligible studies were excluded if they: 1) were duplicates, 2) did not assess HRQoL in the CVI population, 3) did not assess the reliability and/or validity of specific HRQoL questionnaires for CVI patients, 4) were review articles, or 5) investigated samples with post-thrombotic syndrome.

### Quality assessment

The methodological quality of the studies included was verified using the Newcastle-Ottawa Scale adapted for cross-sectional studies,^26^ as recommended by the Cochrane Collaboration. The scale was developed by the Universities of Newcastle, Australia, and Ottawa, Canada, and comprises 8 items grouped under 3 topics, namely, selection, comparability and confounders, and outcome. For the quality assessment, a scoring criterion from zero to 10 stars was used, grouped into 3 items: selection, comparability, and outcome. The higher the number of stars, the higher the methodological quality of the study. The maximum score for “Selection” is five stars, the maximum score for “Comparability” is two stars, and the score for “Outcome” can be a maximum of three stars. For the risk of bias assessment, studies that scored in all domains (selection, comparability, and outcome) were classified as high quality.^27^ Those that did not score in at least one of the domains were rated as low quality. All of the studies found in the electronic search were included in the review, regardless of methodological quality.

### Outcomes and data analysis

The following data were extracted from the articles included in this review: authors, year of publication, sample characteristics (sample size, age, CEAP class, percentage of women), HRQoL questionnaires used, psychometric properties (reliability and validity), and methodological quality. The primary outcomes were those related to reliability and validity. If any of these data were missing, the study’s corresponding author was contacted.

Internal consistency and inter-rater and intra-rater repeatability were considered as measures of reliability. Internal consistency indicates whether all subparts of an instrument measure the same characteristic and is generally verified using Cronbach’s alpha coefficient.^28^ Cronbach’s alpha coefficient reflects the degree of covariance between items on a scale. Repeatability measures the degree to which similar results are obtained at two different times, i.e., it is the estimate of the stability of the measures.^29^ Repeatability is evaluated using intraclass correlation coefficients (ICC) (continuous variables) or the Kappa index (categorical variables). Values equal to or greater than 0.70 were considered adequate for Cronbach’s alpha coefficients, ICCs, and the Kappa index.^20^^,^^30^^,^^31^

Validity was evaluated with (1) the correlation between the specific questionnaire score and scores on the SF-36 (criterion validity) or other HRQoL questionnaires (construct validity), and (2) hypothesis testing (construct validity), based on examining the differences in scores between samples with different levels of disease severity. Coefficients above 0.5 for correlations between the specific questionnaire and another standard questionnaire were considered adequate.^32^ For hypothesis tests, the questionnaire was classified as adequate when scores were statistically different (p<0.05) between different CEAP classes.

## RESULTS

**Figure 1:**
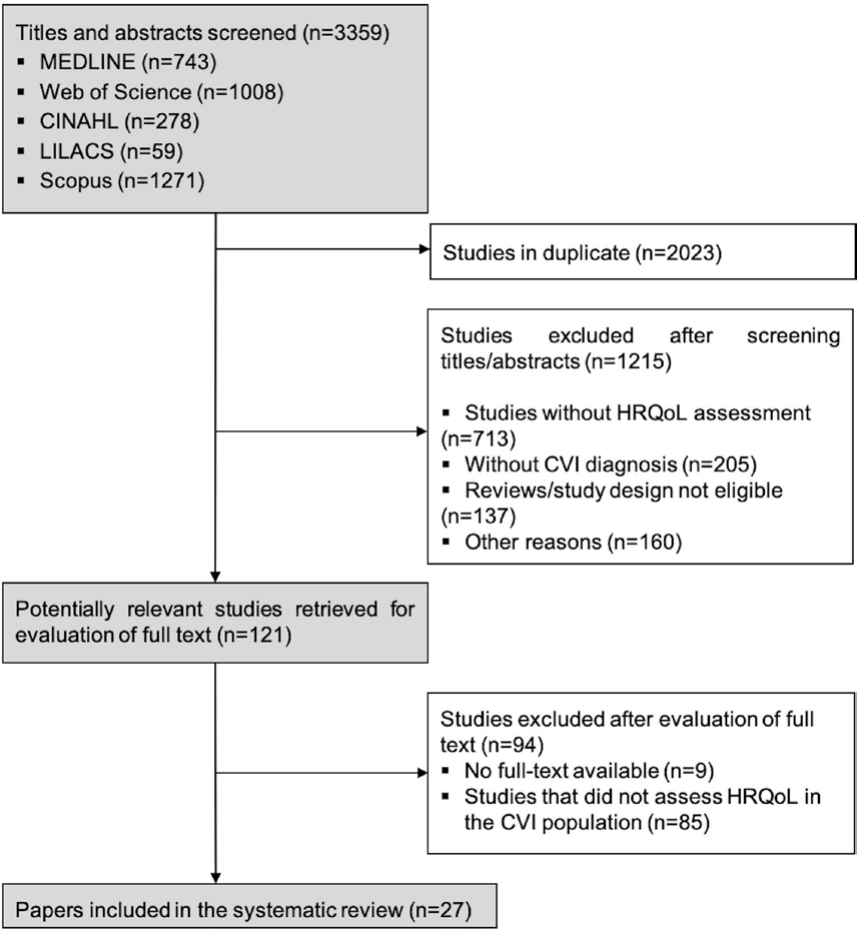
Flow of studies through the review. MEDLINE: Medical Literature Analysis and Retrieval System Online; CINAHL: Cumulative Index to Nursing and Allied Health Literature; LILACS: Latin American & Caribbean Health Sciences Literature.

The flow of studies through the review is illustrated in Figure 1. Initial research identified 3,359 studies, 2,023 (60.2%) of which were duplicates. After screening titles and abstracts, 1,215 papers were excluded. Most of them were reviews, studies that did not assess HRQoL in patients with CVI, or studies that did not verify the psychometric properties of the questionnaires. A further 94 studies were excluded after reading the full texts and a total of 27 papers were included in the present review.

### HRQoL questionnaires

Seven different questionnaires were identified in the 27 studies included. Seven studies^33^^-^39 used the Chronic Lower Limb Venous Insufficiency Questionnaire (CIVIQ) and its adaptations (CIVIQ-14 and CIVIQ-20), seven^40^^-^46 used the Venous Insufficiency Epidemiological and Economic Study - Quality of life/Symptom (VEINES-QoL/Sym) instrument, five^47^^-^51 used the Aberdeen Varicose Vein Questionnaire (AVVQ), and one study^52^ used the Swedish version of a short patient-reported outcome measure for superficial venous insufficiency (VARIShort). Two ulcer-specific questionnaires were administered in studies that included patients with venous leg ulcers. Two studies^53^^,^^54^ used the Venous Leg Ulcer Quality of Life questionnaire (VLU-QoL) and five^55^^-^59 used the Charing Cross Venous Ulcer questionnaire (CCVUQ).

### Chronic Lower Limb Venous Insufficiency Questionnaire (CIVIQ)

Four studies that verified the psychometric properties of the CIVIQ-20 (n=6,776) and three that assessed the CIVIQ-14 (n=6,092) were included in this review. The mean quality score for studies assessing the CIVIQ-20 was 5.8 (ranging from 4 to 7) and two of these studies were of high quality and three were of low quality. The mean quality score for studies assessing the CIVIQ-14 was 6.3 (ranging from 5 to 8) and all three were of high quality. The sample details, reliability, and validity properties for the studies assessing the CIVIQ-20 and CIVIQ-14 are shown in Table 1.

**Table 1:** Characteristics of included studies that verified the reliability and validity of the CIVIQ-20 and CIVIQ-14 (n=7).

**Study**	**Version**	**Sample**	**Reliability**		**Validity**		**Score for quality**‡	**Overall quality**
			**Internal consistency**	**Repeatability**	**Hypothesis testing**	**Criterion/construct validity**		
**CIVIQ-20**								
Launois et al.^35^	Original version, in French	n=2,001 patients (CEAP 0 to 4)	(+) for the psychological (α=0.90), physical (α=0.83), and pain dimensions (α=0.83)	Not by ICC	(+) Differences in the global index between patients with and without arteritis (p<0.001)	(?)	Selection (★★★)	Low quality
			(-) for social dimension (α=0.67)				Comparability (-)	
							Outcome (★★)	
							**Total score**: 05/10	
Launois et al.^34^	Multicenter study (18 countries) in several languages	n=4,048 patients (CEAP 0 to 4)	(+) for the global index (α=0.94)	Not by ICC	(+) Differences in the global index and in all dimensions among CEAP 0 to 4 (p<0.001)	(?)	Selection (★★★★)	High quality
			(+) for all dimensions (α=0.86 for physical, 0.89 for psychological, 0.83 for pain, and 0.76 for the social dimension)				Comparability (★)	
							Outcome (★★)	
							**Total score**: 07/10	
Biemans et al.^36^	Dutch version	n=159 patients (CEAP 1 to 6, 53±13.13 years, 70.4% females)	(+) for the global index (α=0.94)	Not by ICC	(+) Differences in the global score among CEAP 0 and 1 versus 2 and 3 versus 4 to 6 (p<0.001)	(+) The correlation with the physical component of the SF-36 was adequate (r= -0.64)	Selection (★★★★)	High quality
						(-) The correlation with the mental component of the SF-36 was not adequate (r= -0.42)	Comparability (★)	
							Outcome (★★)	
							**Total score**: 07/10	
Ozdemir et al.^37^	Turkish population	n=140 patients (CEAP 3 to 6, 52.3±13.40 years, 61.4% females)	(+) for the global index (α=0.93)	(+) for the global index / inter-rater (ICC=0.80)	(?)	(+) The correlation with VEINES-QoL was adequate (r= -0.57; p < 0.001)	Selection (★★★★)	Low quality
							Comparability (-)	
							Outcome (★★)	
							**Total score**: 06/10	
Sinozic et al.^38^	Croatian population	n=428 patients (CEAP 1 to 6, median age of 52 years, 78% females)	(+) for the global index (α=0.94)	(?)	(?)	(?)	Selection (★★)	Low quality
							Comparability (-)	
							Outcome (★★)	
							**Total score**: 04/10	
**CIVIQ-14**								
Launois et al.^33^	Original version confirmed in Polish, Czech, Spanish, and French populations	n=3,004 patients in a multicenter study	(?)	(+) for all dimensions / inter-rater (ICC from 0.88 to 0.94)	(?)	(?)	Selection (★★★)	High quality
				(+) for all items / inter-rater (weighted kappa from 0.81 to 0.87)			Comparability (★)	
							Outcome (★★)	
							**Total score**: 06/10	
Radak et al.^39^	Serbian version	n=2,660 patients (CEAP 0 to 6, 57.4±12.9 years, 72.5% females)	(+) for the global index (α=0.93) and for all dimensions (α from 0.78 to 0.91)	(?)	(+) Differences in the CIVIQ-14 score among CEAP 0 to 6 (p<0.01).	(?)	Selection (★★★)	High quality
							Comparability (★)	
							Outcome (★★★)	
							**Total score**: 08/10	
Sinozic et al.^38^	Croatian version	n=428 patients (CEAP 1 to 6, median age of 52 years, 78% females)	(+) for the global index (α=0.92)	(?)	(+) Differences in the CIVIQ-14 score among CEAP 1 and 2 versus 3 and 4 versus 5 and 6 (p<0.001)	(?)	Selection (★★)	High quality
							Comparability (★)	
							Outcome (★★)	
							**Total score**: 05/10	

Abbreviations: CEAP = Clinical Etiological Anatomical Pathophysiological; CIVIQ = Chronic Lower Limb Venous Insufficiency Questionnaire; SF-36 = Short-form of Health Survey; VEINES: Venous Insufficiency Epidemiological and Economic Study questionnaire; α = Cronbach’s alpha; ICC = intra-class correlation coefficient; r = correlation coefficient.

‡ The stars indicate the quality scores evaluated with the Newcastle-Ottawa scale for cross-sectional studies, ranging from zero to 10 stars. The higher the number of stars, the higher the methodological quality of the study. The maximum score for the “Selection” item is five stars, the maximum score for the “Comparability” item is two stars, and the maximum score for the “Outcome” item is three stars.

(+) classified as adequate according to COnsensus-based Standards for the selection of health status Measurement INstruments (COSMIN); (-) classified as not adequate according to COSMIN; (?) not verified.

The CIVIQ-20 is a disease-specific questionnaire developed in French and validated by Launois and colleagues,^35^ although an English version is also presented in the manuscript. The questionnaire was designed to verify the HRQoL of patients with CVI and CEAP classes from 0 to 4. The instrument comprises 20 items in 4 dimensions (physical, psychological, and social aspects and pain). The higher the score, the worse the patient’s HRQoL. The original version had adequate internal consistency for the psychological, physical, and pain dimensions (Cronbach's α ranging from 0.83 to 0.90), but values for the social dimension were inadequate (Cronbach's α=0.67). Reproducibility was reported using the correlations between the scores, thus, the ICC was not calculated. Hypothesis testing demonstrated a higher score in CVI patients with versus without arteritis (p<0.001).

In a multicenter study conducted in 18 countries,^34^ the CIVIQ-20 showed adequate internal consistency (Cronbach's α=0.94) and the questionnaire was effective in discriminating levels of CVI severity.

Three cross-cultural adaptations^36^^-^38 of the CIVIQ-20 verified the reliability and validity of the questionnaire and, despite including patients with venous leg ulcers, all of them showed adequate internal consistency of the global index (Cronbach's α ranging from 0.93 to 0.94). The Turkish adaptation^37^ also found adequate inter-rater repeatability (ICC=0.80) and construct validity for assessment of HRQoL when compared to VEINES-QoL (r= -0.574; p <0.001). Finally, the Dutch version^36^ seems to be a valid questionnaire to evaluate the physical aspects of HRQoL in patients with CVI (r= -0.64 when compared to the physical component of the SF-36), but not the mental aspects (r= -0.42 when compared to the mental component of the SF-36).

The CIVIQ-14 is a shorter version of the CIVIQ-20 and was described in 2011 by Launois et al.,^33^ aiming to obtain a more stable questionnaire. This new version comprised 14 items in 3 dimensions (physical aspects, psychological aspects, and pain). The original version, tested in several languages, showed adequate test-retest reliability in all domains (ICC ranging from 0.88 to 0.94). The validity of the questionnaire was not verified by comparison with a standard questionnaire, but by correlations between the CIVIQ-14 score and signs of disease severity (cramps, heavy legs, sensation of swelling, and pain).

Two cross-cultural adaptations were found^38^^,^^39^ and both Serbian and Croatian versions showed adequate internal consistency (Cronbach's α of 0.93 and 0.94, respectively). Hypothesis testing showed that the scores for both cross-cultural adaptations were able to discriminate HRQoL between different CEAP classes (p<0.01 for both).

### Venous Insufficiency Epidemiological and Economic Study (VEINES)

The results of the seven studies included that evaluated the reliability and validity of VEINES are shown in Table 2. The mean score for quality was 5.7 (ranging from 3 to 6) and six were of high quality.

**Table 2:** Characteristics of included studies that verified the reliability and validity of VEINES (n=7).

**Study**	**Version**	**Sample**	**Reliability**		**Validity**		**Score for quality**‡	**Overall quality**
			**Internal consistency**	**Repeatability**	**Hypothesis testing**	**Criterion / construct validity**		
Lamping et al.^40^	Original version (English-speaking sample)	n=88 patients (CEAP 1 to 4, 47.5±12.1 years, 92% females)	(+) for VEINES-QoL (α=0.91)	(+) inter-rater (ICC 14 to 30 days: 0.89)	-	(+) The correlation between the score and the physical component of the SF-36 was adequate (r=0.61)	Selection (★★★★)	High quality
						(-) The correlation between the score and the mental (r=0.19) component of the SF-36 was not adequate.	Comparability (★)	
	Original version (French-speaking sample)	n=305 patients (CEAP 0 to 6, 20 to 75 years, 81% females)	(+) for VEINES-QoL (α=0.88 to 0.92)	(+) inter-rater (ICC 14 to 30 days: 0.89)	-	(+) The correlation between the score and the physical (r=0.52 to 0.69) component of the SF-36 was adequate.	Outcome (★★)	
						(-) The correlation between the score and mental (r=0.34 to 0.49) component of the SF-36 was not adequate (r=0.34 to 0.49).	**Total score**: 07/10	
	Original version (Italian-speaking sample)	n=143 patients (CEAP 0 to 6, 53.1±12.7 years, 78% females)	(+) for VEINES-QoL (α=0.94)	-	-	(+) The correlations between the VEINES-QoL score and the physical (r=0.73) and mental (r=0.55) components of the SF-36 were adequate.		
	Original version (Canadian French -speaking sample)	n=79 patients (CEAP 0 to 6, 45.5±11.8 years, 92% females)	(+) for VEINES-QoL (α=0.90)	-	-	(+) The correlation between the VEINES-QoL score and the physical (r=0.71) component of the SF-36 was adequate		
						(-) The correlation between the VEINES-QoL score and the mental (r=0.26) component of the SF-36 was not adequate		
Kutlu et al.^41^	Turkish adaptation	n=118 hospitalized patients (CEAP 1 to 5, 44.37±1.71 years, 74.6% females)	(+) for VEINES-QoL (α=0.86)		(+) Differences in VEINES- QoL (p<0.001) between CEAP classes	(+) The correlations between the VEINES-QoL and the SF-36 domains physical functioning (r=0.66), role physical (r=0.57), pain (r=0.60), vitality (r=0.50), social functioning (r=0.56), and role emotional (r=0.50) were adequate	Selection (★★★)	High quality
						(-) The correlations between the VEINES-QoL and the SF-36 domains general health (r=0.48), and mental health (r=0.43) were not adequate	Comparability (★)	
							Outcome (★★)	
							**Total score**: 06/10	
Tuygun et al.^42^	Turkish adaptation	n=100 patients (CEAP not reported, 41.9±12.5 years, 68% females)	(+) for the global index (α=0.91)	(+) inter-rater (ICC=0.99; n=30)	-	(+) The correlations between the VEINES-QoL score and the physical (r=0.78) and mental (r=0.65) components of the SF-36 were adequate	Selection (★★★)	High quality
							Comparability (★)	
							Outcome (★★)	
							**Total score**: 06/10	
van der Velden et al.^43^	Dutch adaptation	n=66 patients (CEAP 1 to 6, 54.9±13.1 years, 73% females)	(+) for VEINES-QoL (α=0.88)	-	(-) No differences in VEINES-QoL between classes C1 versus C2 versus C3 to C6 (p=0.41)	(+) The correlation between the VEINES-QoL score and the physical component of the SF-36 was adequate (r=0.6)	Selection (★)	Low quality
						(-) The correlation between the VEINES-QoL score and the mental component of SF-36 was not adequate (r=0.2)	Comparability (-)	
							Outcome (★★)	
							**Total score**: 03/10	
Bland et al.^44^	Patients with venous leg ulcers	n=450 patients with venous leg ulcers (68.6±14.5 years, 49.3% females)	(+) for VEINES-QoL (α=0.88)	(+) inter-rater (ICC=0.80)	-	(+) The correlations between the VEINES-QoL score and the physical (r=0.58, p<0.001) and mental (r=0.58, p<0.001) components of the SF12 were adequate	Selection (★★★)	High quality
							Comparability (★)	
							Outcome (★★)	
							**Total score**: 06/10	
Sinabulya et al.^45^	Swedish adaptation	n=112 patients (CEAP 1 to 6, a majority at CEAP 2, 54.5±15.2 years, 75% females)	(+) for VEINES-QoL (α=0.93)	-	(-) No differences in VEINES-QoL (p=0.22) between CEAP classes	(+) The correlations between the VEINES-QoL and the SF-36 domains bodily pain (r=0.82), general health (r=0.55), physical functioning (r=0.70), role emotion (r=0.57), role physical (r=0.61), social functioning (r=0.54) and vitality (r=0.54) were adequate	Selection (★★★)	High quality
						(-) The correlation between the VEINES-QoL and the SF-36 domain mental health (r=0.45) was not adequate	Comparability (★)	
							Outcome (★★)	
							**Total score**: 06/10	
Ribeiro-Samora et al.^46^	Brazilian adaptation	n=98 patients (CEAP 1 to 6, 60.73±14.11 years, 88.8% females)	-	-	(-) No differences in VEINES-Sym (p=0.70) or VEINES-QoL (p=0.40) between CEAP 1, 2, and 3 versus 4, 5, and 6.	-	Selection (★★★)	High quality
							Comparability (★)	
							Outcome (★★)	
							**Total score**: 06/10	

Abbreviations: CEAP = Clinical Etiological Anatomical Pathophysiological; SF-36 = Short-form of Health Survey; VEINES = Venous Insufficiency Epidemiological and Economic Study questionnaire; α = Cronbach’s alpha; ICC = intra-class correlation coefficient; r = correlation coefficient.

‡ The stars indicate the quality scores evaluated with the Newcastle-Ottawa scale for cross-sectional studies, ranging from zero to 10 stars. The higher the number of stars, the higher the methodological quality of the study. The maximum score for the “Selection” item is five stars, the maximum score for the “Comparability” item is two stars, and the maximum score for the “Outcome” item is three stars.

(+) classified as adequate according to COnsensus-based Standards for the selection of health status Measurement INstruments (COSMIN); (-) classified as not adequate according to COSMIN; (?) not verified.

The VEINES-QoL/Sym was developed by Lamping and colleagues from the United Kingdom, Belgium, and Canada,^40^ and first administered in English, French, Italian, and Canadian French. The instrument consists of 26 items related to symptoms (10 items), limitations in daily activity (9 items), the hour of the day with the highest intensity of symptoms (1 item), changes in HRQoL over the last year (1 item) and the psychological impact caused by the disease (5 items). Two scores are generated from the questionnaire: the VEINES-QoL related to the HRQoL, and the VEINES-Sym, related to the presence of symptoms. Twenty-five of the 26 items in the questionnaire are used to calculate the VEINES-QoL. For VEINES-Sym, only a subgroup of the 10 items related to symptoms are included, all of them present in VEINES-QoL. Thus, only the VEINES-QoL results were included in the review. For analysis of scores, higher values indicate better outcomes.

The original questionnaire^40^ showed adequate internal consistency in all languages. It also showed adequate reproducibility in the languages in which it was evaluated, that is, English and French. Regarding validity, only the questionnaire in the Italian language showed an adequate correlation with both the physical and mental components of the SF-36. In English, French, and Canadian French, the VEINES-QoL had an adequate correlation with the physical component of the SF-36, but an inadequate correlation with its mental component.

In a multicenter study with CVI patients with venous leg ulcers conducted in England and Northern Ireland,^44^ the VEINES-QoL showed adequate internal consistency, reproducibility, and validity according to comparisons with both physical and mental components of the SF-12.

Six cross-cultural adaptations that evaluated the reliability and validity of the VEINES-QoL were found: two in Turkish populations,^41^^,^^42^ two in Swedish populations,^45^^,^^52^ one in a Dutch population,^43^ and one in a Brazilian^46^ population. Internal consistency was adequate in all five versions in which it was evaluated (it was not evaluated in the Brazilian adaptation). Reproducibility was adequate in the two versions in which it was evaluated.^42^^,^^52^ The results regarding validity were heterogeneous. In general, the score showed an inadequate correlation with the mental component or with the mental health domain of the SF-36, except for in a study by Tuygun et al.^42^ In this study, the validity was adequate for both the physical and mental components. Additionally, three studies^43^^,^^45^^,^^46^ found no significant differences between CEAP classes, while just one^41^ showed a significant difference between classes.

### Aberdeen Varicose Vein Questionnaire (AVVQ)

Five studies were found that investigated the reliability or validity of the AVVQ. The mean score for quality was 6.8 (ranging from 6 to 7) and four articles were of high quality. The results of these studies are shown in Table 3.

**Table 3:** Characteristics of included studies that verified the reliability and validity of the AVVQ (n=5).

**Study**	**Version**	**Sample**	**Reliability**		**Validity**		**Score for quality**‡	**Overall quality**
			**Internal consistency**	**Repeatability**	**Hypothesis testing**	**Criterion / construct validity**		
Garrat et al.^51^	Original version, in English	n=281 patients with varicose veins (45±8 years, 76% females)	(+) for the global index (α=0.72)	(?)	(?)	(-) The correlations with the SF-36 domains were not adequate [physical functioning (r= -0.49), role physical (r= -0.35), social functioning (r= -0.44), role emotional (r= -0.41), mental health (r= - 0.31), vitality (r= -0.38), pain (r= -0.44), and general health (r= -0.25)]	Selection (★★★★)	High quality
							Comparability (★)	
							Outcome (★★)	
							**Total score**:07/10	
Smith et al.^47^	In English	n=137 patients with varicose veins without ulcerations (median age of 46 years, ranging from 22 to 82, 71% females)	(+) for the global index (α=0.74)	(?)	(?)	(-) The correlations with the SF-36 domains were not adequate [physical functioning (r= -0.39), role physical (r= -0.41), social functioning (r= -0.39), and pain (r= -0.39)]	Selection (★★★★)	Low quality
							Comparability (-)	
							Outcome (★★)	
							**Total score**:06/10	
Klem et al.^48^	Dutch population	n=143 patients (CEAP 1 to 6, 54±13 years, 69% females)	(+) for the global index (α=0.76)	-	(+) The score was significantly different (p<0.01) among CEAP classes (CEAP 1 and 2 versus 3 and 4 versus 5 and 6)	(?)	Selection (★★★★)	High quality
							Comparability (★)	
							Outcome (★★)	
							**Total score**:07/10	
Leal et al.^50^	Brazilian population	n=107 patients (CEAP 1 to 6, 50.1±12,7 years, 87.9% females)	(+) for dysfunction (α=0.77), and esthetic appearance (α=0.73)	(+) inter-rater (ICC=0.95)	(+) The score was significantly different (p=0.012) between CEAP 1, 2, and 3 versus 4, 5, and 6).	(+) The correlations with the SF-36 domains physical functioning (r= -0.51), role physical (r= -0.52), and pain (r= -0.55) were adequate	Selection (★★★★)	High quality
			(-) for the global index (α=0.54), for varicose extension (α=0.64), and for complications (α=0.29)	(+) intra-rater (ICC=0.85)		(-) The correlations with the SF-36 domains social functioning (r= -0.48), role emotional (r= -0.41), mental health (r= - 0.31), vitality (r= -0.28), and general health (r= - 0.32) were not adequate	Comparability (★)	
							Outcome (★★)	
							**Total score**:07/10	
Klem et al.^49^	Dutch population	n=143 patients (CEAP 1 to 6, 54±13 years, 69% females)	(?)	Not by ICC	(+) The score was significantly different (p<0.01) among CEAP classes (CEAP 1 and 2 versus 3 and 4 versus 5 and 6)	(+) The correlation with the SF-36 physical functioning domain (r= -0.54) was adequate	Selection (★★★★)	High quality
						(-) The correlations with the SF-36 domains role physical (r= -0.45), social functioning (r= -0.41), and pain (r= -0.44) were not adequate.	Comparability (★)	
							Outcome (★★)	
							**Total score**:07/10	

Abbreviations: CEAP = Clinical Etiological Anatomical Pathophysiological; SF-36 = Short-form of Health Survey; α = Cronbach’s alpha; ICC = intra-class correlation coefficient; r = correlation coefficient.

‡ The stars indicate the quality scores evaluated with the Newcastle-Ottawa scale for cross-sectional studies, ranging from zero to 10 stars. The higher the number of stars, the higher the methodological quality of the study. The maximum score for the “Selection” item is five stars, the maximum score for the “Comparability” item is two stars, and the maximum score for the “Outcome” item is three stars.

(+) classified as adequate according to COnsensus-based Standards for the selection of health status Measurement INstruments (COSMIN); (-) classified as not adequate according to COSMIN; (?) not verified

The Aberdeen Varicose Vein Questionnaire (AVVQ) is a disease-specific questionnaire that assesses HRQoL in patients with varicose veins. It was developed in Scotland in 1993 by Garratt et al.^51^ Briefly, the 13 items evaluate dimensions related to pain, use of analgesics, social issues, and interference caused by varicose veins at work, household chores, and leisure. The higher the score, the worse the patient’s HRQoL.

The questionnaire was initially administered to 281 patients with varicose veins and demonstrated adequate, but borderline, internal consistency (Cronbach's α=0.72). Additionally, when compared with the SF-36, the score of the original AVVQ version had inadequate measures of validity (weak to moderate correlations with the SF-36 domains; r values from -0.25 to -0.49). Using the same questionnaire in English in the United Kingdom, Smith et al.^47^ also found an adequate and borderline internal consistency (Cronbach's α=0.74). In addition, the questionnaire was not valid for assessment of patients’ HRQoL, since the score showed weak correlations with all SF-36 domains (all correlation coefficients were below 0.5).

This questionnaire was culturally adapted for the Dutch and Brazilian populations.^48^^-^50 The Dutch adaptation demonstrated adequate (but also borderline) internal consistency,^49^ the ability to discriminate different levels of disease severity (CEAP 1 and 2 versus 3 and 4 versus 5 and 6; p<0.01),^49^ and weak to moderate correlation with the SF-36 domains, especially those related to physical aspects.^48^ In the test-retest analysis, a significant and strong association between two scores (2-week interval) was reported (r=0.87, p<0.01), with no difference between them (p=0.12).^49^ The ICC value was not reported.

The Brazilian adaptation^50^ showed no internal consistency for the global index (Cronbach's α=0.54), or for the domains varicose extension (Cronbach's α=0.64) and complications (Cronbach's α=0.29). The Brazilian version also demonstrated adequate intra-rater (ICC = 0.85) and inter-rater (ICC = 0.95) reproducibility and effectiveness for discriminating different levels of severity, but demonstrated weak to moderate correlations with SF-36 domains. It is noteworthy that all cross-cultural adaptations included patients in CEAP classes 1 to 6.

### VARIShort

The Swedish version of a short patient-reported outcome for superficial venous insufficiency (VARIShort) is a questionnaire developed by Hultman and colleagues^52^ to assess HRQoL in patients with superficial venous insufficiency (SVI), considered a “short” version of the VEINES-QoL/Sym. The authors report that the VEINES-QoL/Sym is not specific for SVI, so there was a need to create an easy and comprehensive patient-report measure. The new Swedish version consists of 7 items, 5 on symptoms, 1 on activity, and 1 on appearance. The original version of the questionnaire, in Swedish, was administered to 525 patients at CEAP classes from C2 to C6, with mean age of 58.3 years, and 59% females. Therefore, the new measurement instrument covers patients who are in CEAP classes 2 or higher.

The questionnaire showed adequate internal consistency (Cronbach's α=0.93) and intra-rater repeatability (ICC=0.93). In addition, the score demonstrated a strong correlation with the VEINES-QoL (r= -0.819; p<0.001). No other study or cross-cultural adaptation assessing the psychometric properties of this questionnaire was found. The quality score for the study was 5 (3 stars for selection, none for comparability, and 2 for outcomes) and it was classified as low quality.

### Questionnaires for patients with venous leg ulcers

Two questionnaires for assessing patients with venous leg ulcers were included: the CCVUQ (five studies) and the VLU-QoL (two studies). The characteristics of these studies are shown in Table 4. The mean score for quality of the CCVUQ studies was 5.2 (ranging from 4 to 7). The mean score for quality of VLU-QoL studies was 4.5 (ranging from 4 to 5). One VLU-QoL study was of high quality and the other was of low quality.

**Table 4:** Characteristics of included studies that verified the reliability and validity of ulcer-specific questionnaires (n=7).

**Study**	**Version**	**Sample**	**Reliability**		**Validity**		**Score for quality**‡	**Overall quality**
			**Internal consistency**	**Repeatability**	**Hypothesis testing**	**Criterion / construct validity**		
**CCVUQ**								
Smith et al.^55^	Original version, in English	n=98 patients with venous leg ulcers (median age was 76 years, 60% females)	(+) for the global index (α=0.93)	(+) intra-rater (ICC=0.84, sample subset)		(+) The correlations with the SF-36 domains were adequate [physical functioning (r= -0.60), role physical (r = -0.55), pain (r = -0.57), general health (r =-0.56), vitality (r = -0.55), social functioning (r = –0.71), role emotional (r = -0.52), and mental health (r = -0.57)]	Selection (★★)	Low quality
							Comparability (-)	
							Outcome (★★)	
							**Total score**:04/10	
Wong et al.^56^	Chinese adaptation	n=100 patients with venous leg ulcers (70±10.7 years, 30% females)	(+) for the global index (α=0.95) and for all domains (α>0.90)	(+) ICC (6 weeks)=0.94	(?)	(?)	Selection (★★★)	High quality
							Comparability (-)	
							Outcome (★★)	
							**Total score**:05/10	
Couto et al.^57^	Brazilian adaptation	n=50 patients with venous leg ulcers (CEAP C5 to C6, 63.2±11.74 years, 80% females)	(+) for the global index (α=0.92)	(+) inter-rater / ICC (30 minutes) = 0.95	(?)	(+) The correlation with the SF-36 domains physical functioning (r= -0.58), pain (r = -0.60), general health (r =-0.66), vitality (r = -0.72), and social functioning (r = –0.54) were adequate	Selection (★★)	Low quality
				(+) intra-rater / ICC (7 to 15 days) = 0.95		(-) The correlations with the domains role physical (r = -0.41), role emotional (r = -0.42), and mental health (r = -0.48) of the SF-36 were not adequate	Comparability (-)	
							Outcome (★★)	
							**Total score**:04/10	
Tafernaberry et al.^58^	Uruguayan adaptation	n=50 patients with venous leg ulcers (63.4 years, ranging from 34 to 84, 52% females)	(+) for the global index (α=0.83)	(?)	(?)	(?)	Selection (★★★★)	High quality
							Comparability (★)	
							Outcome (★★)	
							**Total score**:07/10	
Amaral et al.^59^	Brazilian adaptation	n=112 patients with venous leg ulcer (61.39±11.86 years, 50% females)	(+) for the global index (α=0.92)	(+) ICC (30 min) = 0.96	(?)	(?)	Selection (★★★★)	Low quality
							Comparability (-)	
							Outcome (★★)	
							**Total score**:06/10	
**VLU-QoL**								
Hareendran et al.^53^	Original version, in English	n=70 patients with venous leg ulcers (74±12 years old, 69% females)	(+) for all domains (α>0.8)	(+) ICC for all domains (48 to 73h): ranging from 0.83 to 0.86	(+) Patients who reported exudate, edema, and ulcer smell had lower scores on the VLU-QoL	(+) The correlation between the activities domain of VLU-QoL and the physical component of the SF-36 was adequate (r= -0.58; p<0.001)	Selection (★★)	High quality
					(-) There was no significant association between VLU-QoL and hyperpigmentation	(-) The correlation between the activities domain of VLU-QoL and the mental component of the SF-36 was not adequate (r= -0.29; p<0.001)	Comparability (★)	
						(-) The correlations between the psychological domain of VLU-QoL and physical (r= -0.39) and mental (r= -0.46) component of SF-36 were not adequate (p<0.001 for both)	Outcome (★★)	
						(-) The correlations between the symptoms distress domain of VLU-QoL and physical (r= -0.41) and mental (r= -0.40) components of SF-36 were not adequate (p<0.001 for both)	**Total score**:05/10	
Araújo et al.^54^	Brazilian adaptation	n=82 patients with healed and active venous leg ulcer (CEAP 5 to 6, 67.3±13.9 years, 68% females)	(+) for the global index (α=0.94), and for all domains (α>0.91)	(+) ICC=0.90 in 30 days	(?)	(?)	Selection (★★)	Low quality
				(+) ICC=0.78 in 60 days			Comparability (-)	
							Outcome (★★)	
							**Total score**:04/10	

Abbreviations: CEAP = Clinical Etiological Anatomical Pathophysiological; SF-36 = Short-form of Health Survey; α = Cronbach’s alpha; ICC = intra-class correlation coefficient; r = correlation coefficient.

‡ The stars indicate the quality scores evaluated with the Newcastle-Ottawa scale for cross-sectional studies, ranging from zero to 10 stars. The higher the number of stars, the higher the methodological quality of the study. The maximum score for the “Selection” item is five stars, the maximum score for the “Comparability” item is two stars, and the maximum score for the “Outcome” item is three stars.

(+) classified as adequate according to COnsensus-based Standards for the selection of health status Measurement INstruments (COSMIN); (-) classified as not adequate according to COSMIN; (?) not verified.

The CCVUQ was developed by Smith et al.^55^ in a London teaching hospital and surrounding community clinics. The venous ulcer-specific questionnaire consists of 21 items, divided into 4 domains related to social interaction, domestic activities, and emotional and esthetic status. The higher the score, the worse the patient’s HRQoL. The questionnaire showed adequate internal consistency and was considered a valid tool for evaluation of HRQoL when compared to SF-36 domains (correlation coefficients ranged from -0.52 to -0.71). The ICC was not evaluated, but the questionnaire seems to be stable due to the strong correlation (r=0.84; p<0.001) between the scores applied at two different times, with no significant difference between the scores (p=0.86). The psychometric properties of the questionnaires were verified in Chinese,^56^ Brazilian,^57^^,^^59^ and Uruguayan^58^ populations. All cross-cultural adaptations showed good internal consistency. Intra-rater reproducibility has also been confirmed in Brazilian^57^^,^^59^ and Chinese^56^ populations. In the Brazilian population, CCVUQ scores had adequate correlations with the SF-36 domains physical functioning, general health, vitality, and social functioning.

The VLU-QoL questionnaire is also designed for patients with venous leg ulcers and was developed by Hareendran et al.^53^ in a multicenter study with four participating centers in the United Kingdom. The 34-item instrument evaluates HRQoL in terms of activities (12 items), psychological aspects (12 items), and symptoms related to venous leg ulcers (10 items). Higher scores represent poorer HRQoL. The original version^53^ was administered to 70 patients with venous leg ulcers and all domains showed adequate internal consistency and reproducibility. Regarding validity, the mental component of the SF-36 did not show an adequate correlation with any of the three domains of the VLU-QoL and the physical component of the SF-36 only did so with the activities domain. In hypothesis testing, patients who reported exudate, edema, and ulcer smell had lower scores on the VLU-QoL, but there was no significant association with hyperpigmentation. The ICC was not reported. The Brazilian cross-cultural adaptation of the questionnaire^54^ also demonstrated adequate internal consistency and good reproducibility.

Table 5 contains summarized results for the reliability and validity of the CVI-specific HRQoL questionnaires included in the present study.

**Table 5:** Summary of results for the reliability and validity of CVI-specific HRQoL questionnaires.

**HRQoL questionnaire**	**Reliability**	**Validity**
CIVIQ-20	**Internal consistency:** α = from 0.93 to 0.94	**Hypothesis testing**: There were differences in the CIVIQ-20 global index between patients with and without arteritis (p<0.001), and in all dimensions among CEAP 0 to 4 (p<0.001).
	**Inter-rater reproducibility**: ICC = 0.80	**Construct validity:** The coefficient for the correlation between the CIVIQ-20 score and: (1) the physical component of the SF-36 was r= -0.64; (2) the mental component of SF-36 was r= -0.42, and (3) the VEINES-QoL score was r= -0.57.
CIVIQ-14	**Internal consistency:** α = from 0.92 to 0.93	**Hypothesis testing**: There were differences in the CIVIQ-14 score among CEAP 0 to 6 (p<0.01).
	**Inter-rater reproducibility**: ICC = from 0.88 to 0.94	
VEINES-QoL	**Internal consistency:** α = from 0.86 to 0.94	**Hypothesis testing:** There were no differences in VEINES-QoL scores among CEAP classes in three studies. There were differences in VEINES-QoL scores among CEAP classes in one study.
	**Inter-rater reproducibility:** ICC = from 0.80 to 0.99	**Construct validity:**
		- The coefficients for the correlation between the VEINES-QoL score and the physical component of the SF-36 ranged from 0.52 to 0.78. The coefficient for the correlation between the VEINES-QoL score and the mental component of the SF-36 ranged from 0.19 to 0.65.
		- The coefficients for the correlation between the VEINES-QoL score and physical and mental component of the SF-12 were both 0.58.
		- The coefficients for the correlations between the VEINES-QoL and the SF-36 domains were: (1) physical functioning: from 0.66 to 0.70; (2) role physical: from 0.57 to 0.61; (3) pain: from 0.60 to 0.82; (4) general health status: from 0.48 to 0.55; (5) vitality: from 0.50 to 0.54; (6) social functioning: from 0.54 to 0.56; (7) role emotional: from 0.50 to 0.57; and (8) mental health: from 0.43 to 0.45.
AVVQ	**Internal consistency:** α = 0.54 to 0.76	**Hypotheses testing:** The score was significantly different (p<0.012) among CEAP classes.
	**Inter-rater reproducibility:** ICC = 0.95	**Construct validity:** The coefficients for the correlations between the AVVQ and the SF-36 domains were: (1) physical functioning: from -0.39 to -0.54 (2) role physical: from -0.35 to -0.52; (3) pain: from -0.39 to -0.55; (4) general health status: from -0.25 to -0.32; (5) vitality: from -0.28 to -0.38; (6) social functioning: from -0.39 to -0.48; (7) role emotional: -0.41; and (8) mental health: -0.31.
	**Intra-rater reproducibility:** ICC = 0.85	
VARIShort	**Internal consistency:** α=0.93	**Construct validity:** The correlation coefficient between the VARIShort score and the VEINES-QoL score was -0.819.
	**Intra-rater reproducibility:** ICC = 0.93	
CCVUQ	**Internal consistency**: α = 0.83 to 0.95	**Construct validity:** The coefficients for the correlations between the CCVUQ and the SF-36 domains were: (1) physical functioning: from -0.58 to -0.60; (2) role physical: from -0.41 to -0.55; (3) pain: from -0.57 to -0.60; (4) general health status: from -0.56 to -0.66; (5) vitality: from -0.55 to -0.72; (6) social functioning: from -0.54 to -0.71; (7) emotional role: from -0.42 to -0.52; and (8) mental health: from -0.48 to -0.57.
VLU-QoL	**Internal consistency**: α > 0.8	**Hypothesis testing:** Patients who reported exudate, edema and ulcer smell had lower VLU-QoL scores but there was no significant association between VLU-QoL and hyperpigmentation.
	**Intra-rater reproducibility**: ICC from 0.78 to 0.90	**Construct validity:**
		- The coefficients for the correlations between the activities domain of the VLU-QoL and the physical and mental components of the SF-36 were -0.58 and -0.29, respectively.
		- The coefficients for the correlations between the psychological domain of the VLU-QoL and the physical and mental components of the SF-36 were -0.39 and -0.46, respectively.
		- The coefficients for the correlations between the symptoms distress domain of the VLU-QoL and physical and mental components of the SF-36 were -0.41 and -0.40, respectively.

Abbreviations: CIVIQ = Chronic Lower Limb Venous Insufficiency Questionnaire; VEINES-QoL = Venous Insufficiency Epidemiological and Economic Study - Quality of life questionnaire; AVVQ = Aberdeen Varicose Vein Questionnaire; VARIShort = Short patient-reported outcome measure for superficial venous insufficiency questionnaire; CCVUQ = Charing Cross Venous Ulcer Questionnaire; VLU-QoL = Venous Leg Ulcer Quality of Life questionnaire; SF-36 = Short-form of Health Survey; α = Cronbach’s alpha; ICC = intra-class correlation coefficient; CEAP = Clinical Etiological Anatomical Pathophysiological.

## DISCUSSION

The present study demonstrated the reliability and validity of specific questionnaires for assessing the HRQoL of patients with CVI. Systematic discussion of psychometric properties has an important clinical impact and we believe that our findings may guide questionnaire choice and support their use in patient follow-up.

The main findings of the present review were that: (1) the CIVIQ-20 and CIVIQ-14 showed good internal consistency in all domains, except for the social domain. Reproducibility was also adequate. However, few studies have addressed their validity; (2) the VEINES-QoL also showed excellent reliability, but the findings on validity are inconclusive. Thus, to date, the CIVIQ-20 or CIVIQ-14 should be used in patients without venous ulcers; (3) the AVVQ is a questionnaire developed for patients with varicose veins and it had low to inadequate internal consistency and weak correlation with the SF-36 domains; (4) reliability and validity of the VARIShort were reported by only one study; and (5) of the questionnaires for patients with venous leg ulcers, the CCVUQ showed adequate internal consistency, reproducibility, and correlations with many domains of the SF. Another questionnaire, the VLU-QoL, proved to be reliable, but its validity must be investigated. Therefore, for patients with venous leg ulcers, the CCVUQ seems to be the most appropriate in terms of reliability and validity.

The CIVIQ-20 is available in the languages of 34 countries,^60^ but its reliability has only been assessed for the original version and four cross-cultural adaptations. In summary, the results suggest that the CIVIQ-20 and CIVIQ-14 have adequate internal consistency, both in the original version and its cross-cultural adaptations. Only the social dimension of CIVIQ-20 demonstrated inadequate internal consistency. The CIVIQ-14 is even more useful because it is shorter and faster to administer, which facilitates use. Another advantage is the large size of the samples in the studies reviewed that used the CIVIQ. Furthermore, a multicenter study^34^ and the Dutch version^36^ showed the CIVIQ-20 was accurate for discriminating disease severity. Similarly, in two cross-cultural adaptations,^38^^,^^39^ the CIVIQ-14 demonstrated adequate ability to discriminate disease stages. On the other hand, reproducibility and validity in comparison with the SF-36 or other HRQoL questionnaires have been explored little. Therefore, our results suggest that the CIVIQ-20 and CIVIQ-14 are consistent and can assist in identifying the magnitude of CVI severity in these patients, but further studies are required to confirm the validity of the questionnaire for assessing the HRQoL of patients with CVI.

The VEINES-QoL is a widely used questionnaire in patients with CVI. Internal consistency and reproducibility were adequate in the studies included in the present review, even in patients with venous leg ulcers. However, there are concerns with validity. Briefly, the VEINES-QoL did not demonstrate an adequate correlation with the mental component of the SF-36. Thus, we hypothesize that the questionnaire may cover more physical than mental aspects of CVI. Previous studies^61^^,^^62^ reported social isolation and depression in patients with CVI and, therefore, the disease’s impact on mental and emotional aspects should be highlighted in questionnaires. Furthermore, three of the studies included^43^^,^^45^^,^^46^ failed to demonstrate the power to discriminate between CVI severity levels, which may indicate a possible limitation of the questionnaire for assessing HRQoL in patients at different stages of the disease. Based on validity, no evidence was found to support use of the VEINES-QoL for assessing the HRQoL of patients with CVI. In comparison, the CIVIQ-20 and CIVIQ-14 have both shown better performance in terms of validity, despite the small number of studies available. Thus, to date, use of CIVIQ-20 or CIVIQ-14 is recommended for assessing quality of life of patients with CVI but without venous ulcers.

The AVVQ questionnaire was designed for patients with varicose veins, mainly for verifying improvements in HRQoL after surgical interventions.^63^^-^65 Some issues regarding the results found by the studies reviewed that used this questionnaire should be highlighted. First, the questionnaire’s internal consistency is doubtful, even in the original article.^51^ Adaptations in English and Dutch^47^^,^^48^ presented low, although adequate, values for internal consistency, while the version adapted for Brazilian Portuguese^50^ was inadequate. Second, the test-retest reproducibility needs to be explored further, since ICC was only calculated for the Brazilian version.^50^ Third, the questionnaire’s validity was not confirmed, since the score was only weakly correlated with the SF-36 domains. Finally, all of the cross-cultural adaptations included patients at different levels of disease severity, not just patients with varicose veins, who are the questionnaire’s target population. One strength of the AVVQ score was the ability to discriminate between levels of disease severity in the Dutch and Brazilian versions.^49^^,^^50^ However, this characteristic is questionable, since the population used in the comparison was not the questionnaire’s target population.

In patients with venous leg ulcers, the original version of the CCVUQ showed adequate internal consistency in United Kingdom, Chinese, Brazilian, and Uruguayan populations. Reproducibility was also adequate, except for the Uruguayan version,^58^ for which it was not assessed. The score of the original version showed an adequate correlation with all SF-36 domains. In the Brazilian population,^57^ the correlation was only not adequate with some domains. Accuracy for discriminating between severity levels was not verified, since all patients had active or healed ulcers (CEAP 5 or 6). Therefore, the questionnaire appears to be consistent, stable, and valid for assessing the HRQoL of patients with venous leg ulcers. One limitation is that three of the five studies included had samples smaller than 100 patients, which is the recommended size for high-quality studies.

The VLU-QoL questionnaire also assesses the HRQoL of patients with venous leg ulcers and, like the CCVUQ, it also proved to be consistent and with adequate reproducibility in the versions in English and Brazilian Portuguese.^53^^,^^54^ However, the validity of the questionnaire was only verified in the English version^53^ and showed inadequate results for two of the three domains. Thus, according to the findings of this review, it is recommended that the CCVUQ should be used for patients with venous leg ulcers, since the psychometric properties of this questionnaire have been verified by a larger number of studies and it has more accurate validity.

The present study has some strengths and limitations. One limitation is that the number of studies assessing the psychometric properties of each questionnaire should have been higher. Additionally, many studies used samples that differed from the questionnaires’ target populations. On the other hand, most of the studies included (66.6%) were classified as high quality in the risk of bias assessment, which makes the result consistent. Additionally, the present study did not only include questionnaires validated for a specific country or adapted for a specific language. Therefore, the present study summarized the results for the reliability and validity of disease-specific questionnaires for assessment of the HRQoL of patients with CVI. Thus, the results should assist health professionals to choose a reliable, valid, and disease-specific questionnaire for patients with CVI, in addition to contributing to future research.

## CONCLUSION

The present study suggests that the CIVIQ-20 and CIVIQ-14 have potential value for assessment of HRQoL in patients with CVI (Cronbach's α ranging from 0.92 to 0.94, ICC greater than 0.8), regardless of the severity of the disease, despite their validity having been little reported. Additionally, the VEINES-QoL seems to be consistent (Cronbach's α ranging from 0.86 to 0.93) and reproducible (ICC greater than 0.8), but its validity is still doubtful. The AVVQ showed inadequate internal consistency (Cronbach's α ranging from 0.54 to 0.76) and validity. Among the questionnaires designed for patients with venous leg ulcers, the CCVUQ emerges as a reliable (Cronbach's α ranging from 0.83 to 0.95, ICC greater than 0.95) and valid tool. However, it is emphasized that all of these questionnaires have important limitations and, therefore, the results must be interpreted with caution.

## References

[1] Eberhardt RT, Raffetto JD (2014). Chronic venous insufficiency. Circulation.

[2] van Uden CJ, van der Vleuten CJ, Kooloos JG, Haenen JH, Wollersheim H (2005). Gait and calf muscle endurance in patients with chronic venous insufficiency. Clin Rehabil.

[3] Cetin C, Serbest MO, Ercan S, Yavuz T, Erdogan A (2016). An evaluation of the lower extremity muscle strength of patients with chronic venous insufficiency. Phlebology.

[4] Santler B, Goerge T (2017). Chronic venous insufficiency - a review of pathophysiology, diagnosis, and treatment. J Dtsch Dermatol Ges.

[5] Lurie F, Passman M, Meisner M (2020). The 2020 update of the CEAP classification system and reporting standards. J Vasc Surg Venous Lymphat Disord.

[6] Rabe E, Berboth G, Pannier F (2016). Epidemiology of chronic venous diseases. Wien Med Wochenschr.

[7] Carlton R, Mallick R, Campbell C, Raju A, O’Donnell T, Eaddy M (2015). Evaluating the expected costs and budget impact of interventional therapies for the treatment of chronic venous disease. Am Health Drug Benefits.

[8] Padberg FT, Johnston MV, Sisto SA (2004). Structured exercise improves calf muscle pump function in chronic venous insufficiency: a randomized trial. J Vasc Surg.

[9] Pieper B, Templin TN, Birk TJ, Kirsner RS (2008). Chronic venous disorders and injection drug use: impact on balance, gait, and walk speed. J Wound Ostomy Continence Nurs.

[10] Branisteanu DE, Feodor T, Baila S, Mitea IA, Vittos O (2018). Impact of chronic venous disease on quality of life: results of vein alarm study. Exp Ther Med.

[11] Alonso J, Ferrer M, Gandek B (2004). Health-related quality of life associated with chronic conditions in eight countries: results from the International Quality of Life Assessment (IQOLA) Project. Qual Life Res.

[12] Del Buono MG, Arena R, Borlaug BA (2019). Exercise intolerance in patients with heart failure: JACC state-of-the-art review. J Am Coll Cardiol.

[13] Lane TR, Sritharan K, Herbert JR, Franklin IJ, Davies AH (2013). Management of chronic venous disease by primary care. Phlebology.

[14] Rocha FA, Lins EM, Almeida CC (2020). Quality of life assessment before and after surgery for lower limb varicose veins. J Vasc Bras.

[15] Silva KLS, Figueiredo EAB, Lopes CP (2021). The impact of exercise training on calf pump function, muscle strength, ankle range of motion, and health-related quality of life in patients with chronic venous insufficiency at different stages of severity: a systematic review. J Vasc Bras.

[16] Silva JL, Lima AG, Diniz NR, Leite JC (2021). Effectiveness of therapeutic exercises for improving the quality of life of patients with chronic venous insufficiency: a systematic review. J Vasc Bras.

[17] Youn YJ, Lee J (2019). Chronic venous insufficiency and varicose veins of the lower extremities. Korean J Intern Med.

[18] Cook DA, Beckman TJ (2006). Current concepts in validity and reliability for psychometric instruments: theory and application. Am J Med.

[19] Pittman J, Bakas T (2010). Measurement and instrument design. J Wound Ostomy Continence Nurs.

[20] Terwee CB, Bot SD, de Boer MR (2007). Quality criteria were proposed for measurement properties of health status questionnaires. J Clin Epidemiol.

[21] Roach KE (2006). Measurement of health outcomes: reliability, validity and responsiveness. J Prosthet Orthot.

[22] Mokkink LB, Terwee CB, Patrick DL (2010). The COSMIN study reached international consensus on taxonomy, terminology, and definitions of measurement properties for health-related patient-reported outcomes. J Clin Epidemiol.

[23] Caggiati A, De Maeseneer M, Cavezzi A, Mosti G, Morrison N (2018). Rehabilitation of patients with venous diseases of the lower limbs: state of the art. Phlebology.

[24] Shamseer L, Moher D, Clarke M (2015). Preferred reporting items for systematic review and meta-analysis protocols (PRISMA-P) 2015: elaboration and explanation. BMJ.

[25] Deeks JJ, Higgins J, Altman DG, Green S (2022). Cochrane handbook for systematic reviews of interventions version 5.1.0.

[26] Wells G, Shea B, O’Connell D, Peterson J, Welch V, Losos M (2022). The Newcastle-Ottawa Scale (NOS) for assessing the quality of nonrandomised studies in meta-analyses.

[27] Lee J, Park JH, Jwa H, Kim YH (2017). Comparison of efficacy of intravenous peramivir and oral oseltamivir for the treatment of influenza: systematic review and meta-analysis. Yonsei Med J.

[28] Streiner DL (2003). Starting at the beginning: an introduction to coefficient alpha and internal consistency. J Pers Assess.

[29] Polit DF, Beck CT (2011). Fundamentos de pesquisa em enfermagem: métodos, avaliação e utilização..

[30] Asunta P, Viholainen H, Ahonen T, Rintala P (2019). Psychometric properties of observational tools for identifying motor difficulties: a systematic review. BMC Pediatr.

[31] Prinsen CAC, Mokkink LB, Bouter LM (2018). COSMIN guideline for systematic reviews of patient-reported outcome measures. Qual Life Res.

[32] Mokkink LB, Prinsen CA, Patrick DL, Alonso J, Bouter LM, de Vet HC (2022). COSMIN methodology for systematic reviews of Patient‐Reported Outcome Measures (PROMs).

[33] Launois R, Le Moine JG, Lozano FS, Mansilha A (2012). Construction and international validation of CIVIQ-14 (a short form of CIVIQ-20), a new questionnaire with a stable factorial structure. Qual Life Res.

[34] Launois R, Mansilha A, Jantet G (2010). International psychometric validation of the Chronic Venous Disease quality of life Questionnaire (CIVIQ-20). Eur J Vasc Endovasc Surg.

[35] Launois R, Reboul-Marty J, Henry B (1996). Construction and validation of a quality of life questionnaire in chronic lower limb venous insufficiency (CIVIQ). Qual Life Res.

[36] Biemans AA, van der Velden SK, Bruijninckx CM, Buth J, Nijsten T (2011). Validation of the chronic venous insufficiency quality of life questionnaire in Dutch patients treated for varicose veins. Eur J Vasc Endovasc Surg.

[37] Ozdemir OC, Tonga E, Tekindal A, Bakar Y (2016). Cross-cultural adaptation, reliability and validity of the Turkish version of the Chronic Venous Disease Quality of Life Questionnaire (CIVIQ-20). Springerplus.

[38] Sinozic T, Bazdaric K, Sverko D, Ruzic A, Katic M (2017). Validation of the Croatian version of CIVIQ quality of life questionnaire in patients with chronic venous disorders. Croat Med J.

[39] Radak DJ, Vlajinac HD, Marinkovic JM, Maksimovic MZ, Maksimovic ZV (2013). Quality of life in chronic venous disease patients measured by short Chronic Venous Disease Quality of Life Questionnaire (CIVIQ-14) in Serbia. J Vasc Surg.

[40] Lamping DL, Schroter S, Kurz X, Kahn SR, Abenhaim L (2003). Evaluation of outcomes in chronic venous disorders of the leg: development of a scientifically rigorous, patient-reported measure of symptoms and quality of life. J Vasc Surg.

[41] Kutlu A, Yilmaz E, Cecen D, Eser E, Ozbakkaloglu A (2011). The Turkish validity and reliability of the venous insufficiency epidemiological and economic study-quality of life/symptoms scales. Angiology.

[42] Tuygun AK, Ketenci B, Gunay R (2012). Validity and reliability of VEINES-QOL/Sym questionnaire in chronic venous disorders. J Cardiovasc Surg.

[43] van der Velden SK, Biemans AA, Nijsten T, Sommer A (2014). Translation and validation of the Dutch VEINES-QOL/Sym in varicose vein patients. Phlebology.

[44] Bland JM, Dumville JC, Ashby RL (2015). Validation of the VEINES-QOL quality of life instrument in venous leg ulcers: repeatability and validity study embedded in a randomised clinical trial. BMC Cardiovasc Disord.

[45] Sinabulya H, Bergstrom G, Hagberg J, Johansson G, Blomgren L (2018). Cultural adaptation and validation of the Swedish VEINES-QOL/Sym in patients with venous insufficiency. Phlebology.

[46] Ribeiro-Samora GA, Carvalho MLV, Moura RMF, Pereira DAG (2019). Limitations of VEINES QOL/SYM for discriminating chronic venous insufficiency severity. J Vasc Bras.

[47] Smith JJ, Garratt AM, Guest M, Greenhalgh RM, Davies AH (1999). Evaluating and improving health-related quality of life in patients with varicose veins. J Vasc Surg.

[48] Klem TM, Sybrandy JE, Wittens CH (2009). Measurement of health-related quality of life with the Dutch translated Aberdeen Varicose Vein Questionnaire before and after treatment. Eur J Vasc Endovasc Surg.

[49] Klem TM, Sybrandy JE, Wittens CH, Bot MLE (2009). Reliability and validity of the Dutch translated Aberdeen Varicose Vein Questionnaire. Eur J Vasc Endovasc Surg.

[50] Leal FJ, Couto RC, Pitta GBB (2015). Validação no Brasil de Questionário de Qualidade de Vida na Doença Venosa Crônica (Questionário Aberdeen para Veias Varicosas no Brasil/AVVQ-Brasil). J Vasc Bras.

[51] Garratt AM, Macdonald LM, Ruta DA, Russell IT, Buckingham JK, Krukowski ZH (1993). Towards measurement of outcome for patients with varicose veins. Qual Health Care.

[52] Hultman KH, Sinabulya H, Blomgren L (2021). Validation of a Swedish version of a short patient-reported outcome measure for superficial venous insufficiency. J Vasc Surg Venous Lymphat Disord.

[53] Hareendran A, Doll H, Wild DJ (2007). The venous leg ulcer quality of life (VLU-QoL) questionnaire: development and psychometric validation. Wound Repair Regen.

[54] Araújo RB, Fortes MR, Abbade LP, Miot HA (2014). Translation, cultural adaptation to Brazil and validation of the venous leg ulcer quality of life questionnaire (VLU-QoL-Br). Rev Assoc Med Bras.

[55] Smith JJ, Guest MG, Greenhalgh RM, Davies AH (2000). Measuring the quality of life in patients with venous ulcers. J Vasc Surg.

[56] Wong IK, Lee DT, Thompson DR (2006). Translation and validation of the Chinese version of the Charing Cross Venous Ulcer Questionnaire. J Clin Nurs.

[57] Couto RC, Leal FJ, Pitta GBB (2016). Validação do questionário de qualidade de vida na úlcera venosa crônica em língua portuguesa. J Vasc Bras.

[58] Tafernaberry G, Otero G, Agorio C, Dapueto JJ (2016). Adaptación y evaluación inicial del Charing Cross Venous Ulcer Questionnaire en pacientes con úlceras venosas crónicas en Uruguay. Rev Med Chil.

[59] Amaral KVA, Melo PG, Alves GR (2019). Charing Cross Venous Ulcer Questionnaire - Brasil: estudo bicêntrico de confiabilidade. Acta Paul Enferm.

[60] Les Laboratoires Servier (2022). CIVIQ users’ guide.

[61] Bergan JJ, Schmid-Schonbein GW, Smith PD, Nicolaides AN, Boisseau MR, Eklof B (2006). Chronic venous disease. N Engl J Med.

[62] van Korlaar IM, Vossen CY, Rosendaal FR (2004). The impact of venous thrombosis on quality of life. Thromb Res.

[63] Chan CY, Chen TC, Hsieh YK, Huang JH (2011). Retrospective comparison of clinical outcomes between endovenous laser and saphenous vein-sparing surgery for treatment of varicose veins. World J Surg.

[64] Mekako AI, Hatfield J, Bryce J, Lee D, McCollum PT, Chetter I (2006). A nonrandomised controlled trial of endovenous laser therapy and surgery in the treatment of varicose veins. Ann Vasc Surg.

[65] Nandhra S, El-sheikha J, Carradice D (2015). A randomized clinical trial of endovenous laser ablation versus conventional surgery for small saphenous varicose veins. J Vasc Surg.

